# Parental Educational Involvement and Academic Delay of Gratification Among Chinese Adolescents: The Chain-Mediating Role of Self-Control and Consideration of Future Consequences

**DOI:** 10.3390/bs16030407

**Published:** 2026-03-11

**Authors:** Ming Zhang, Yifei Li, Hui Zhao

**Affiliations:** Faculty of Education, Henan Normal University, Xinxiang 453007, China; zhangming@stu.htu.edu.cn (M.Z.);

**Keywords:** parental educational involvement, academic delay of gratification, self-control, consideration of future consequences, junior school students

## Abstract

Parental educational involvement is a pivotal factor associated with an individual’s academic development; however, its specific association with academic delay of gratification and the underlying mechanisms remain not fully understood. Drawing upon ecosystem theory, this study examined a serial mediation model to explore the links between parental educational involvement and academic delay of gratification, focusing on the mediating roles of self-control and consideration of future consequences. A cross-sectional design was employed, involving 726 junior high school students. Data were collected using the Parental Educational Involvement Scale, Academic Delay of Gratification Scale, Self-Control Scale, and Consideration of Future Consequences Scale, with statistical analyses performed via SPSS 26.0 and the PROCESS macro. The results indicated that: (1) parental educational involvement, academic delay of gratification, self-control, and consideration of future consequences were positively correlated with each other in a pairwise manner; (2) academic delay of gratification was significantly predicted by parental educational involvement (*β* = 0.117, *p* < 0.001); (3) self-control and consideration of future consequences play a mediating role in the relationship between parental education involvement and academic delay of gratification. There are three mediating pathways involved in this process: the individual mediating role of self-control (*β* = 0.092, 95% CI [0.054, 0.133]), the individual mediating role of consideration of future consequences (*β* = 0.030, 95% CI [0.015, 0.050]) and the chain mediating role of self-control and consideration of future consequences (*β* = 0.015, 95% CI [0.008, 0.024]). This study examined the internal mechanism between parental educational involvement and academic delay of gratification, which is helpful in improving junior middle school students’ ability to engage in academic delay of gratification.

## 1. Introduction

In the contemporary era of digitalization and intelligence, novel forms of temptation—such as short-video addiction and gamified instant feedback—emerge incessantly. These digital distractions are closely associated with short-sighted behaviors in adolescents, potentially complicating their ability to resist impulses for immediate gratification (H. Chen et al., 2025; Xu et al., 2024). Empirical evidence among Chinese university students indicates that short videos, general internet activities, and online gaming account for 43.9%, 35.3%, and 20.8% of online behaviors, respectively (Xia et al., 2023), representing potential challenges to sustained academic development. Given that academic achievement is a protracted process rather than a short-term sprint, adolescents must exert consistent volitional effort and effective self-regulation to forgo immediate temptations in favor of long-term objectives.

Academic delay of gratification represents a domain-specific extension of the broader delay of gratification construct within education. Since its systematic introduction by Bembenutty and Karabenick (1998), the conceptualization of this construct has expanded significantly. From a self-regulation perspective, academic delay of gratification is viewed as a rational choice wherein learners weigh the utility of immediate impulses against long-term academic rewards (Bembenutty & Karabenick, 2013). Conversely, through the lens of impulse control, it is defined as the active inhibition of immediate psychological urges to secure distal goals (D. Chen & Chen, 2013). Within the specific context of the Chinese educational environment, this study adopts the definition proposed by X. D. Li (2005): academic delay of gratification is the psychological tendency of students in competitive academic settings to voluntarily forgo tempting activities that satisfy immediate needs in pursuit of learning goals with greater long-term value. The extant literature suggests that academic delay of gratification is positively linked to self-regulated learning abilities and negatively associated with depressive symptoms, thereby contributing to overall mental health (Y. Li, 2024; Ye et al., 2025).

Junior high school (ages 12–15) represents a critical developmental transition from childhood to adolescence (Piaget, 2008), rendering the exploration of academic delay of gratification particularly salient. On one hand, students in this cohort experience a developmental imbalance between cognitive processing and emotional regulation. Specifically, the prefrontal cortex’s capacity for inhibitory control remains in a state of maturation (Casey et al., 2008), which may be associated with a heightened susceptibility to immediate gratification when confronted with temptations. On the other hand, the increasing complexity of academic curricula and the impending pressure of the Senior High School Entrance Examination significantly extend the feedback cycles for learning tasks. This academic context requires individuals to forgo short-term leisure in favor of distal academic achievement (H. L. Li & Zhang, 2014). Notably, while existing research indicates that cognitive abilities generally advance with age, some students exhibit a marked regression in their academic delay of gratification capacity during higher grades (J. Liu et al., 2018). Given this context, investigating the factors associated with junior high school students’ academic delay of gratification is of substantial practical significance.

According to Bronfenbrenner’s (1979) ecosystem theory, individual development emerges from the interaction between the individual and multi-layered environmental systems. Within this framework, the microsystem—representing the environment with the most proximal and frequent contact—plays a foundational role in shaping developmental outcomes, distinct from more distal layers such as the mesosystem or macrosystem. Family interaction is central to the microsystem, with parental educational involvement serving as a critical component. This involvement reflects not only external environmental support but also the process by which such support is internalized into individual motivation. Existing literature has established that parental educational involvement is positively associated with adolescents’ academic development, cognitive enhancement, and mental health (Z. Yang & Zhang, 2025), while being negatively linked to problem behaviors and academic anxiety (Cheng et al., 2019). However, its specific relationship with academic delay of gratification remains under-explored; thus, this study focuses on these family-level microsystem factors to elucidate this association.

Furthermore, Self-Regulation Theory posits that the attainment of goals depends on a complex regulatory system comprising an executive volitional level and a motivational evaluation level (Hall & Fong, 2007). Self-control, as a volitional effort, reflects the capacity to inhibit immediate impulses in favor of goal-directed behavior (Duckworth & Steinberg, 2015). Conversely, consideration of future consequences, as a motivational orientation, reflects an individual’s valuation and preference regarding cross-temporal goals (Murphy & Dockray, 2018). This theoretical perspective provides a robust framework for understanding how parental educational involvement may be internalized into individual behavioral tendencies. Specifically, positive parental involvement has been identified as a predictor of higher self-control levels through the provision of structured support (Saeed et al., 2023). Individuals with higher self-control often demonstrate superior cognitive monitoring, which is associated with a greater focus on the distal consequences of their behavior(Watson & Milfont, 2017). Concurrently, learners with a high consideration of future consequences typically exhibit more adaptive learning states and a stronger tendency toward academic delay of gratification (Y. Z. Yang & Zeng, 2022). Consequently, self-control and consideration of future consequences may serve as mediating mechanisms between parental educational involvement and academic delay of gratification.

However, there is currently a lack of empirical research systematically exploring the direct effect of parental educational involvement on academic delay of gratification, and few studies have revealed the mediating pathways of self-control and consideration of future consequences in this relationship. Based on this, the present study attempts to integrate ecosystem theory and self-regulation theory to construct a serial mediation model to elucidate the relationship between parental educational involvement, as an external environmental factor, and academic delay of gratification. Meanwhile, social control theory and social cognitive theory are introduced as complementary perspectives to further explore the potential mediating roles of self-control and consideration of future consequences, thereby constructing a comprehensive model from external ecological resources to internal volitional and motivational processing. The findings will not only contribute to deepening the understanding of the underlying mechanisms of academic delay of gratification among junior high school students but also provide targeted empirical support for precision intervention practices for adolescents.

### 1.1. The Relationship Between Parental Educational Involvement and Academic Delay of Gratification

The concept of parental educational involvement, originating in the United States, has received extensive academic attention. It encompasses the collective behaviors, beliefs, and developmental expectations parents adopt across home and school settings to support their children’s academic and psychological growth. Structurally, this construct typically includes three core dimensions: behavioral, cognitive, and affective involvement (Seginer, 2006; Hu et al., 2023). High-quality involvement is further characterized by six key features: structure, autonomy support, a focus on the learning process, positive emotional engagement, consistent joint involvement of both parents, and the child’s receptive perception of such support (L. Luo et al., 2014). Academic delay of gratification, as a domain-specific manifestation of self-regulation, involves the strategic allocation of psychological energy and volitional monitoring to secure distal academic goals (L. Chen & Yeung, 2025).

Crucially, this complex psychological function does not develop in isolation. Drawing on ecosystem theory (Bronfenbrenner, 2000), an individual’s psychological functioning is shaped through continuous interaction with their proximal environment. As the primary microsystem for adolescents, the family represents a foundational environment linked to students’ cognitive, behavioral, and social development through daily interactions and the transmission of values (F. Chen et al., 2024). Specifically, when parents provide high levels of autonomy support and process-focused attention, adolescents are more likely to internalize academic requirements as intrinsic values. Such internalization is associated with a greater capacity to mobilize volitional strategies to resist immediate temptations, such as entertainment or leisure, in favor of long-term objectives (Holzer et al., 2024). Furthermore, research indicates that emotional warmth from Chinese parents is positively associated with the development of adolescent self-discipline, providing an emotional foundation for academic delay of gratification (Q. Liu & Wang, 2021). At the cognitive level, parental educational expectations are significantly correlated with self-regulation capacity and the effective use of cognitive strategies (J. Li et al., 2024). Consequently, it is hypothesized that parental educational involvement, as a targeted environmental resource, serves as a significant predictor of students’ capacity for academic delay of gratification.

It is essential to acknowledge that parental educational involvement within the Chinese cultural context diverges significantly from Western paradigms (Wang, 2023). Influenced by Confucian traditions, Chinese-style parenting integrates “strictness” and “love” into the indigenous construct of Guan (to govern and to care). Unlike Western perspectives, which often dichotomize autonomy support and psychological control, Chinese parents frequently regard high demands and rigorous behavioral monitoring as manifestations of emotional warmth and parental responsibility. Under this dual “pressure-support” system, Chinese adolescents generally do not perceive intensive parental involvement as a threat to their autonomy; rather, they tend to internalize such involvement as a supportive resource that is associated with enhanced resilience and self-discipline. This cultural nuance provides a unique psychological framework wherein adolescents may be more inclined to suppress immediate impulses in pursuit of distal academic excellence. Based on these considerations, the present study proposes the following hypothesis:

**H1:**  *Parental educational involvement is positively correlated with academic delay of gratification.*

### 1.2. The Mediating Role of Self-Control

Self-control is defined as an individual’s capacity to actively make adaptive adjustments to their psychological states and behaviors to achieve goals in the absence of external supervision (Duckworth, 2011). The association between parental educational involvement and adolescent self-control can be understood as a socialization process wherein external social constraints are internalized as psychological qualities. According to Social Control Theory, an individual’s impulse inhibition and normative behavior depend heavily on the social bonds established within their microsystem, particularly the family (Wiatrowski, 1978). Specifically, parental educational involvement facilitates an informal social control system through dimensions such as parent–child attachment and shared value systems. When parents demonstrate high levels of academic concern, emotional support, and consistent behavioral norms, these distal expectations are internalized—through continuous proximal interactions—into stable psychological functions (Sun et al., 2022; van der Veen et al., 2025). In other words, high-quality parental involvement strengthens adolescents’ identification with and belonging to the family value system, potentially prompting them to mobilize cognitive resources to inhibit immediate impulses that conflict with academic objectives. Notably, while longitudinal evidence (Ferreira et al., 2018) has identified initial levels of parental educational involvement as significant predictors of subsequent self-control, the present study focuses on the concurrent association between these constructs. It is therefore inferred that parental educational involvement is closely linked to the development of adolescent self-control through the internalization of social bonds.

Furthermore, according to self-regulation theory, behavioral regulation encompasses core components such as standard setting, process monitoring, and volitional execution (Zimmerman, 2000). Within this framework, self-control serves as a central volitional function, associated with the inhibition of immediate impulses arising from the limbic system. Academic delay of gratification represents the domain-specific application of these volitional resources. Fundamentally, it reflects a decision-making tendency wherein individuals mobilize volitional resources to prioritize high-value objectives when faced with conflicts between immediate pleasure and distal academic achievement (Bembenutty, 2007). Empirical evidence consistently indicates that both internal and external control are closely associated with delayed gratification. Specifically, individuals with higher levels of self-control tend to exhibit a stronger capacity for academic delay of gratification (C. Zhang et al., 2016). It is therefore conjectured that those with greater self-control are more adept at diverting attention from immediate temptations and maintaining a sustained focus on distal academic goals. Based on the preceding discussion, this study proposes the following hypothesis:

**H2:**  *Self-control mediates the relationship between parental educational involvement and academic delay of gratification.*

### 1.3. The Mediating Role of Consideration of Future Consequences

Consideration of future consequences refers to the degree to which individuals weigh the potential distant outcomes of their current behaviors and the extent to which they are influenced by those outcomes (Strathman et al., 1994). Higher levels of this construct indicate a greater focus on the delayed consequences of behavior, whereas lower levels suggest a predominant focus on immediate outcomes (Hevey et al., 2010). Subsequent research has bifurcated this construct into two dimensions: consideration of future consequences—future; consideration of future consequences—immediate. This is a two-dimensional model with established reliability and validity (Joireman et al., 2012). According to expectancy-value theory, motivation to engage in specific activities is linked to an individual’s expectations for success and the subjective value assigned to those activities (Wigfield & Eccles, 2000). Within this framework, as adolescents internalize aspirations for long-term development, their consideration of future consequences tends to increase. Empirical evidence suggests that high-quality parental educational involvement—characterized by concrete academic guidance and a supportive environment—is positively associated with the development of positive future expectations in adolescents, reflecting more forward-looking orientations in their current behavioral choices (Liang et al., 2020; Dou et al., 2022). Consequently, it is hypothesized that parental educational involvement serves as a positive predictor of consideration of future consequences.

Furthermore, the Sensitization Hypothesis and the Buffering Hypothesis offer a robust theoretical framework for understanding the association between consideration of future consequences and academic delay of gratification (Joireman et al., 2008). The Sensitization Hypothesis posits that individuals with a high consideration of future consequences possess a heightened cognitive sensitivity to distal outcomes. This sensitivity may facilitate the translation of academic planning and long-term expectations—often internalized through parental educational involvement—into concrete behavioral motivations. Conversely, the Buffering Hypothesis suggests that consideration of future consequences serves as a psychological resource that moderates the perceived attractiveness of immediate temptations, such as leisure or social impulses. This buffering effect is associated with stronger defensive regulation when individuals confront academic conflicts. Extant empirical research indicates that consideration of future consequences, by strengthening affective and cognitive connections to future events, is negatively associated with a preference for immediate rewards. Such a focus relates to a greater tendency among students to prioritize distal goals in their academic planning (Y. Z. Yang & Zeng, 2022). Consequently, a positive relationship is expected between consideration of future consequences and academic delay of gratification. Based on the preceding discussion, this study proposes the following hypothesis:

**H3:**  *Consideration of future consequences mediates the relationship between parental educational involvement and academic delay of gratification.*

### 1.4. The Chain Mediating Role of Self-Control and Consideration of Future Consequences

Self-control and consideration of future consequences may function as serial mediators between parental educational involvement and academic delay of gratification. According to Social Cognitive Theory, individual functioning arises from the triadic reciprocal determinism of environmental factors, internal cognitions, and behavioral patterns (Bandura, 2001). As a pivotal microsystem, parental educational involvement not only shapes behavioral paradigms through role modeling and the transmission of expectations but is also associated with enhanced self-regulatory efficacy by optimizing metacognitive processes such as goal setting (Kok et al., 2022). From the perspective of the resource model of self-regulation, individuals with high self-control can effectively mitigate cognitive interference from immediate impulses, thereby preserving cognitive resources for distal mental simulations. Empirical evidence supports this logic: Robson et al. (2020) noted that individuals with superior self-regulation capacities, due to their ability to manage immediate distractions, tend to develop clearer and more positive expectations regarding the distal consequences of their behavior. Furthermore, when adolescents can accurately represent and value distal academic rewards, these cognitive assessments are linked to increased motivational reserves for academic delay of gratification. Conversely, a deficit in self-control may render individuals more susceptible to immediate reinforcement, which is associated with a weakened psychological connection to long-term goals and lower outcome expectations (Barnett et al., 2019). Consequently, parental educational involvement may foster the psychological capacity to contemplate distal outcomes by strengthening self-control, which in turn relates to a higher consideration of future consequences and a stronger tendency toward academic delay of gratification. Based on the aforementioned theoretical framework, this study proposes the following hypothesis:

**H4:**  *Self-control and consideration of future consequences play a chain mediating role between parental educational involvement and academic delay of gratification.*

**Figure 1:**
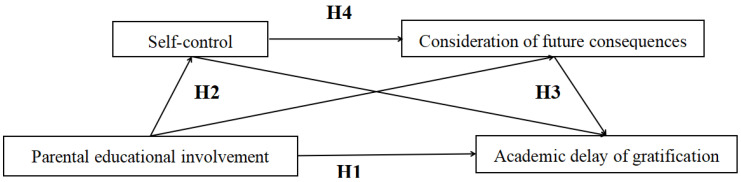
The chain mediation effect model of parental educational involvement and academic delay of gratification.

Based on the foregoing discussion, this study, grounded in ecological systems theory and other relevant theoretical frameworks, selected junior high school students as the research subjects and constructed a chain mediation model to examine the relationship between parental educational involvement and academic delay of gratification, as well as the underlying mechanisms. The model is illustrated in Figure 1.

## 2. Methods

### 2.1. Participants

To empirically examine the conceptual model and the hypothesized mediating pathways, this study employed a cross-sectional design to investigate the associations between parental educational involvement and academic delay of gratification, as well as the mediating roles of self-control and consideration of future consequences. A survey was conducted among 800 students from a key secondary school in Zhengzhou City, Henan Province, China. While the single-school sampling approach may limit the generalizability of the findings, the school’s location in a province characterized by intense educational competition provides a highly representative context for observing the variables of interest. After excluding 74 invalid questionnaires—specifically those characterized by systematic response bias (e.g., patterned or identical answers) or significant missing data—a total of 726 valid responses were retained, yielding an effective response rate of 91%. The participants ranged in age from 12 to 16 years, with a mean age of 13.1 years. The sample consisted of 343 males (47.2%) and 383 females (52.8%). Regarding grade distribution, 243 students were in Grade 7 (33.5%; 117 males, 126 females), 194 students were in Grade 8 (26.7%; 88 males, 106 females), and 289 students were in Grade 9 (39.8%; 138 males, 151 females).

The research protocol received formal approval from the Ethics Committee of the authors’ affiliated institution. Throughout the data collection process, the study strictly adhered to the principle of dual informed consent. First, written informed consent was obtained from the students’ guardians through forms sent home for review and signature. Subsequently, prior to the formal administration of the survey, the examiners provided a comprehensive explanation of the research objectives and the principles of anonymity and confidentiality to the minor participants, securing their unanimous verbal assent. All questionnaires were distributed and collected based on the voluntary participation and full awareness of the students, their parents, and the school faculty.

### 2.2. Research Instruments

#### 2.2.1. Parental Educational Involvement Scale

The Parental Educational Involvement Scale, developed by Song (2010), was employed in this study. The scale consists of 21 items categorized into three dimensions: emotional involvement (e.g., “My parents encourage me when my exam results are not ideal”), intellectual involvement (e.g., “My parents study textbooks or supplementary materials themselves to help tutor me”), and behavioral management involvement (e.g., “My parents manage the time I spend online or using mobile phones”). Participants rated their perceived level of parental educational involvement using a 4-point Likert scale. The total score for parental educational involvement was calculated as the mean of all items, with higher scores indicating more significant parental involvement. In the present study, the Cronbach’s α for the scale was 0.894, indicating excellent internal consistency. Specifically, the Cronbach’s α for emotional involvement was 0.884; for intellectual involvement, it was 0.810; and for behavioral management involvement, it was 0.798. Confirmatory factor analysis (CFA) results indicated sound structural validity ($x^{2}/df\;$= 3.17, CFI = 0.91, TLI = 0.89, RMSEA = 0.06). While the scale comprises three dimensions, empirical research within the Chinese cultural context has demonstrated that the total score robustly represents the overall level of parental involvement perceived by children (C. L. Liu et al., 2018).

#### 2.2.2. Academic Delay of Gratification Scale

The Academic Delay of Gratification Scale for primary and secondary school students, revised by X. D. Li (2005), was utilized in this study. The scale consists of nine situational scenarios, each offering students two contrasting choices. For instance, in Scenario 1, the choices are: (A) “Playing with peers whenever I have free time and cramming just before exams,” or (B) “Playing with peers only after completing all learning tasks.” For each scenario, four response options are provided (e.g., “Definitely choose A,” “Probably choose A,” “Probably choose B,” and “Definitely choose B”). Participants were asked to select the one option that best reflected their actual thoughts. A 4-point Likert scale was used for scoring. The final score for academic delay of gratification was calculated as the mean score of all items, with higher scores indicating a higher capacity for academic delay of gratification. This scale has demonstrated good reliability and validity in the Chinese context (Zhao et al., 2023). In the present study, the Cronbach’s α coefficient for this scale was 0.787, indicating acceptable reliability. CFA results indicated sound structural validity ($x^{2}/df$ = 2.52 CFI = 0.97, TLI = 0.95, RMSEA = 0.05).

#### 2.2.3. Self-Control Scale

The Brief Self-Control Scale, developed by T. Luo et al. (2021), was used in this study. The scale consists of seven items, including three positively scored items (e.g., “I am good at resisting temptation”) and four reverse-scored items (e.g., “I sometimes do things that give me pleasure but are harmful to me”). A 5-point Likert scale was employed for scoring. The self-control score was calculated as the mean score of all items, with higher scores representing a higher level of self-control. This scale has demonstrated good reliability and validity in China (Zuo et al., 2023). In the present study, the Cronbach’s α coefficient for this scale was 0.718, indicating acceptable internal consistency. CFA results indicated sound structural validity ($x^{2}/df$= 1.55, CFI = 0.99, TLI = 0.98, RMSEA = 0.03).

#### 2.2.4. Consideration of Future Consequences Scale

The Consideration of Future Consequences Scale, revised by Ji et al. (2013), was utilized in this study. The scale consists of 12 items, including five positively scored items (e.g., “I consider future issues and try to address them bit by bit in my daily life”) and seven reverse-scored items (e.g., “I only take actions to solve current problems and do not think much about the future”). A 5-point Likert scale was employed for scoring. The total score for consideration of future consequences was calculated as the mean of all items, with higher scores indicating a stronger inclination toward future consequences. This scale has demonstrated good reliability and validity in the Chinese context (Y. P. Liu et al., 2019). In the present study, the Cronbach’s α coefficient for this scale was 0.712, indicating acceptable reliability. CFA results indicated sound structural validity ($x^{2}/df$= 2.92, CFI = 0.93, TLI = 0.90, RMSEA = 0.05).

### 2.3. Procedure and Data Analysis

Data collection was conducted through collective administration on a class-by-class basis. Headteachers read the instructions aloud, and participants were required to complete the questionnaires independently within a designated 20 min period according to their actual thoughts. All questionnaires were collected on-site immediately upon completion. Upon completion of data collection, the raw data were entered by two research assistants. To ensure accuracy, the study implemented a double-entry verification procedure, and a third researcher conducted random spot checks and cross-references with the original questionnaires. These measures were taken to minimize manual entry errors to the greatest extent possible and to safeguard the reliability of the data (Barchard & Pace, 2011). Statistical analysis was performed using SPSS 26.0, which included a common method bias test, descriptive statistics, and correlation analysis. Subsequently, mediation effect analysis was conducted using the PROCESS macro for SPSS, developed by Hayes (2017).

## 3. Results

### 3.1. Common Method Bias Test

As all variables were collected via self-report measures, the potential for common method bias was addressed through both procedural and statistical controls. Procedurally, complete anonymity was guaranteed during data collection, and social desirability bias was minimized via a rigorous informed consent process. Statistically, Harman’s single-factor test was employed for preliminary screening (H. Zhou & Long, 2004). While acknowledging the sensitivity limitations of this approach, it remains a widely utilized diagnostic tool in empirical psychological research. The results identified 13 factors with eigenvalues greater than 1; crucially, the first factor accounted for only 17.214% of the total variance, well below the established 40% threshold. These findings suggest that common method bias is unlikely to compromise the interpretability of the current results.

Furthermore, multicollinearity diagnostics were conducted to ensure the robustness of the regression model. The Variance Inflation Factors (VIF) for all predictor variables ranged from 1.073 to 1.258, and all Tolerance values exceeded 0.795. These indicators fall within acceptable ranges, confirming the absence of severe multicollinearity and satisfying the statistical prerequisites for the subsequent mediation analysis.

### 3.2. Descriptive Statistics and Correlation Analysis

Descriptive statistics (means and standard deviations) and Pearson correlation analysis were conducted for all variables. Since sex and grade are categorical variables, they were converted into dummy variables for analysis: sex was coded as “0 = male, 1 = female”; Grade ^1^ was coded as “1 = Grade 8,” and Grade ^2^ was coded as “1 = Grade 9.” As shown in Table 1, parental educational involvement, academic delay of gratification, self-control, and consideration of future consequences were all significantly and positively correlated with each other.

### 3.3. Hypothesis Testing

To examine the direct predictive effect of parental educational involvement on academic delay of gratification, as well as the serial mediating roles of self-control and consideration of future consequences. The PROCESS macro (Model 6) in SPSS 26.0 was employed to test the proposed model. Model 6 was selected because it is specifically designed to verify serial mediation effects, allowing for the simultaneous estimation and comparison of the significance of multiple indirect paths. This aligns with the current study’s serial mediation analysis regarding how “parental involvement affects delay of gratification through self-control and future expectations.” Furthermore, given the sound reliability of the scales, the impact of measurement error on regression estimates remains limited; thus, the Bootstrap procedure provided by PROCESS (5000 samples) is sufficient to ensure the robustness of the effect estimates. Furthermore, prior to conducting the regression analysis and testing the mediation effects, all continuous variables (e.g., parental educational involvement) were standardized using Z-scores. The regression analysis results (presented in Table 2) indicate the following: First, parental educational involvement significantly and positively predicts self-control (*β* = 0.202, *p* < 0.001). When parental educational involvement and self-control were simultaneously entered to predict consideration of future consequences, both factors exhibited significant predictive effects (*β* = 0.159, *p* < 0.001 and *β* = 0.392, *p* < 0.001, respectively). Subsequently, when parental educational involvement, self-control, and consideration of future consequences were all included in the regression equation to predict academic delay of gratification, the results showed that parental educational involvement (*β* = 0.117, *p* < 0.001), self-control (*β* = 0.458, *p* < 0.001), and consideration of future consequences (*β* = 0.191, *p* < 0.001) each had a significant positive predictive effect on academic delay of gratification.

The bias-corrected percentile Bootstrap procedure (with 5000 resamples) was employed to test the mediation effects (see Table 3). The results indicated that the total indirect effect of the mediation model was 0.138, with a 95% confidence interval (CI) of [0.094, 0.183]. As the interval does not include zero, the mediation effect in the model is significant. Specifically, the indirect effect comprises three pathways: Indirect Effect 1 via self-control (*β* = 0.092, 95% CI [0.054, 0.133]), Indirect Effect 2 via consideration of future consequences (*β* = 0.030, 95% CI [0.015, 0.050]), and the serial mediation effect—Indirect Effect 3—via both self-control and consideration of future consequences (*β* = 0.015, 95% CI [0.008, 0.024]). These three pathways accounted for 36.08%, 11.76%, and 5.88% of the total effect, respectively. Furthermore, the direct effect of parental educational involvement on academic delay of gratification was significant (*β* = 0.117, 95% CI [0.057, 0.178]), indicating that self-control and consideration of future consequences play partial mediating roles in the model.

**Figure 2:**
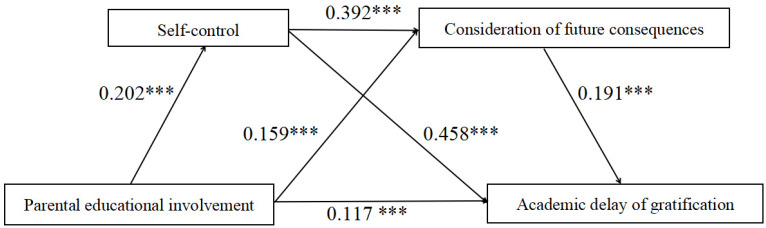
Schematic of chain mediation between parental educational involvement and academic delay of gratification. Note: *** *p* < 0.001.

Although all hypothesized paths reached statistical significance, a further analysis of the effect sizes revealed differences in the relative weights of the various paths in explaining the model. Specifically, the direct effect of parental educational involvement on academic delay of gratification accounted for the largest proportion, indicating its independent and dominant predictive role. Among the three indirect paths, the individual mediating effect of self-control was the most prominent, whereas consideration of future consequences and the chain mediating effect of both provided relatively less explanatory power. Nonetheless, it should be noted that these proportional estimates are sample-specific and reflect only the circumstances of the specific sample in this study (Figure 2).

## 4. Discussion

The results of this study demonstrate a significant positive relationship between parental educational involvement and academic delay of gratification among junior high school students. Specifically, higher levels of parental educational involvement are closely associated with a greater capacity for academic delay of gratification, a finding that aligns with previous literature on the link between parental support and academic achievement (M. Li et al., 2022); thus, Hypothesis 1 was supported. In the current model, the direct path between parental educational involvement and academic delay of gratification accounts for 45.88% of the total effect. This underscores that in a digital era characterized by ubiquitous immediate temptations, high-quality parental involvement functions as a vital external ecological resource for maintaining students’ academic focus. This association can be elucidated through Ecological Systems Theory, which posits that individual development occurs through dynamic interactions within multi-layered environments. Proximal systems, such as the family, are particularly predictive of an individual’s coping mechanisms when confronted with stress or temptation. Consistent with prior research (Hill & Tyson, 2009; Đurišić & Bunijevac, 2017), parental educational involvement represents a foundational environmental support that is linked to children’s psychological functioning. For instance, by providing emotional warmth and a structured growth environment, high-quality involvement may facilitate the development of adaptive study habits and enhance academic self-efficacy through autonomous exploration (X. H. Zhou et al., 2023). When adolescents internalize this external support, they are more likely to exhibit robust self-regulation in the face of immediate distractions, thereby prioritizing distal academic objectives. Consequently, parental educational involvement serves as a significant predictor of academic delay of gratification in junior high school students, highlighting its essential role within the academic microsystem.

The findings also reveal that self-control mediates the relationship between parental educational involvement and academic delay of gratification, providing empirical support for Hypothesis 2. Notably, the statistical results indicate that the predictive power of self-control on academic delay of gratification is significantly stronger than the direct predictive power of parental educational involvement. This underscores the central importance of internal volitional qualities in the delay of gratification process. Specifically, adolescents’ perception of external parental support is positively associated with their cognitive regulation and self-control levels, which in turn are linked to superior academic outcomes—a finding consistent with previous literature (Yao et al., 2024; Shi & Ma, 2021). Furthermore, the mediating effect of self-control accounted for 36.08% of the total effect. For junior high school students, whose prefrontal cortex (responsible for inhibitory control) is still maturing, the internalization of parental support into self-regulatory capacity appears to be a particularly critical pathway. From a theoretical perspective, the socialization process within the family environment is closely linked to the development of an individual’s self-control. According to Self-Regulation Theory (Mithaug, 1993), self-control resources are dynamic rather than static. Parental educational involvement may provide a secure emotional foundation and reduce internal psychological friction, thereby allowing more cognitive resources to be allocated toward self-monitoring and impulse inhibition. Within the specific context of Chinese family culture, moderate parental control and high expectations are often interpreted by adolescents as manifestations of care and responsibility. This positive mental representation may be associated with enhanced self-control motivation following the perception of parental involvement (W. Zhang & Wang, 2023). Higher self-control reflects clear self-awareness, enabling learners to implement effective regulation across cognitive, emotional, and behavioral domains, which is correlated with robust social adaptability (Y. Y. Zhang et al., 2021). Consequently, these individuals are more likely to minimize maladaptive behaviors and focus on distal personal development. Therefore, the mediating role of self-control between parental educational involvement and academic delay of gratification is well-supported.

The results further indicate that consideration of future consequences mediates the relationship between parental educational involvement and academic delay of gratification, consistent with previous findings linking external support to future-oriented cognition (Ramos-Vera et al., 2024); thus, Hypothesis 3 was supported. These findings extend the application of Expectancy-Value Theory in several ways. First, the results suggest that adolescents’ consideration of future consequences is associated with the dual influence of individual traits and environmental contexts. Within this framework, the tendency to engage in specific activities is linked to an individual’s expectations and the subjective value assigned to those activities. Specifically, as adolescents internalize aspirations for distal development, their consideration of future consequences tends to increase. Second, consistent with Locke et al. (2016), high levels of parental involvement are closely related to adolescents’ future well-being, as a supportive socio-ecological environment may facilitate reflective thinking regarding distal goals (Johnson et al., 2014). Finally, students who maintain positive expectations for future outcomes are more likely to engage in proactive academic planning. This capacity to align current actions with distal academic objectives represents a core manifestation of academic delay of gratification (Zu & Lu, 2015; Zu et al., 2021). Consequently, individuals perceiving robust educational support from their parents may be better equipped to adopt a positive orientation toward their distal academic development. By emphasizing the long-term benefits of their behavioral choices, these students exhibit a higher propensity for academic delay of gratification.

The results indicate that the positive association between parental educational involvement and academic delay of gratification among junior high school students is partially explained through a serial mediation path involving self-control and consideration of future consequences, supporting Hypothesis 4. This finding aligns with the core tenets of Ecological Systems Theory, which posits that high-quality interactions within the microsystem serve as essential correlates of an individual’s psychological development and behavioral patterns. Specifically, robust behavioral support from parents (Çelik, 2024) not only provides a clear external framework for adolescents but also facilitates the internalization of external rules through positive feedback mechanisms. This process may support a transition from heteronomy to autonomy. Building upon this, individuals with higher levels of self-control are likely to more acutely perceive the intrinsic links between current behaviors and distal outcomes (J. Li et al., 2021). Consequently, when confronted with immediate temptations, they are more inclined to regulate their impulses and prioritize academic goals with distal value. It is this positive anticipation and systematic consideration of future consequences that relates to adaptive decision-making regarding academic delay of gratification. These findings suggest a sequential process where proximal environmental support is internalized into volitional capacity, which subsequently refines future-oriented cognition to foster academic persistence.

### 4.1. Practical Implications

First, the findings confirm that parental educational involvement is a positive predictor of academic delay of gratification among junior high school students. This suggests that parents can foster a supportive developmental environment through high-quality parent–child interactions. It is recommended that parents serve as proactive role models in daily life and engage in the collaborative development of long-term academic plans with their children. Such strategies may facilitate the internalization of external family support into children’s sustained academic persistence.

Second, the pivotal mediating role of self-control suggests that enhancing an individual’s self-regulatory capacity is associated with a stronger capacity for academic delay of gratification. Educators should collaboratively monitor students’ psychological states, providing timely support during periods of “self-control fatigue.” By teaching students evidence-based self-regulation strategies, educators can help them maintain focus on distal goals when confronted with immediate temptations.

Third, based on the path analysis, consideration of future consequences serves as a vital bridge linking parental support to academic behavior. Educators could guide students to deepen their understanding of the connection between current behavioral choices and future development through targeted group counseling activities, such as writing a “Letter to My Future Self.” Such interventions may assist students in constructing a clear distal vision and enhancing their affective commitment, thereby fostering a greater propensity to postpone immediate gratification in pursuit of long-term objectives.

### 4.2. Limitations and Future Directions

Despite its contributions, several limitations of this study warrant consideration. First, the cross-sectional design poses inherent challenges to internal validity and causal inference. As all variables were assessed at a single time point, this study cannot definitively establish temporal precedence. Specifically, the observed associations may be subject to reverse causality; for instance, adolescents with higher inherent self-control may exhibit adaptive academic behaviors that subsequently elicit more proactive parental educational involvement. Consequently, the mediation pathways identified here should be interpreted as predictive models based on statistical associations rather than confirmed causal mechanisms. Future research should employ longitudinal designs or cross-lagged panel models to more rigorously delineate these developmental trajectories.

Second, the data relied exclusively on student self-reports. Although Harman’s single-factor test suggested that common method bias was within an acceptable range, the risk of inflated correlation coefficients remains, as all variables were rated by the same respondents. This design might amplify the reported strength of associations. To enhance data precision and mitigate potential bias, future studies should incorporate multi-informant data from both parents and children.

Third, the sample was limited to a single key secondary school in Henan Province. Given that Henan is characterized by intense academic competition, and parents in key schools often exhibit higher-than-average parental educational involvement, this specific context may have magnified the observed predictive effects. Therefore, the generalizability of these findings to regions with different educational resource distributions or lower competitive pressures requires further verification. Future research should expand the sampling scope to include diverse provinces and various school types (e.g., ordinary secondary schools or rural schools).

Fourth, this study focused on only two mediating variables: self-control and consideration of future consequences. While these are critical, other psychological mechanisms—such as time management disposition or achievement motivation—may also mediate the link between parental involvement and academic delay of gratification. Investigating these additional factors would provide a more comprehensive understanding of the underlying mechanisms.

Finally, regarding research instruments, the Academic Delay of Gratification Scale utilized a forced-choice structure. As noted by the reviewers, this design may induce social desirability bias, prompting participants to favor options aligned with academic norms over their genuine behavioral tendencies. This measurement constraint may affect the precision of the behavioral estimates. Future research could employ situational experiments to conduct more objective and ecologically valid evaluations.

## 5. Conclusions

The current study systematically examined the association between parental educational involvement and academic delay of gratification among junior high school students, along with its underlying mechanisms. After controlling for demographic variables such as sex, grade, and age, the results confirmed that parental educational involvement is not only a significant predictor of academic delay of gratification but is also linked to it through the serial mediating roles of self-control and consideration of future consequences. The model demonstrated robust statistical power and explanatory depth, offering a valuable theoretical framework for understanding the complex links between parental involvement and adolescents’ capacity for delay of gratification. These findings suggest that future initiatives could focus on developing and validating integrated intervention strategies. Such efforts should aim to systematically foster academic delay of gratification by enhancing parental educational involvement, strengthening adolescents’ self-control, and clarifying their consideration of future consequences.

## Figures and Tables

**Table 1:** Descriptive Statistics and Correlation Matrix of Each Variable.

Variable	*M*	*SD*	1	2	3	4	5	6	7	8
1. Sex	0.528	0.500	1							
2. Age	13.065	0.869	−0.003	1						
3. Grade 1	0.267	0.443	0.023	−0.041	1					
4. Grade 2	0.398	0.490	−0.008	0.885 ***	−0.491 ***	1				
5. PEI	2.886	0.586	−0.082 *	0.030	0.026	0.021	1			
6. SC	3.552	0.690	0.015	−0.067	−0.067	−0.042	0.198 ***	1		
7. CFC	3.462	0.526	−0.026	−0.051	−0.051	−0.050	0.238 ***	0.425 ***	1	
8. ADG	3.269	0.566	0.119 **	−0.001	0.007	0.000	0.244 ***	0.526 ***	0.409 ***	1

Note: * *p* < 0.05, ** *p* < 0.01, *** *p* < 0.001. PEI, parental educational involvement; SC, Self-Control; CFC, consideration of future consequences; ADG, Academic delay of gratification.

**Table 2:** Regression Analysis Among Variables.

Variable	SC			CFC			ADG		
	*β*	*SE*	*t*	*β*	*SE*	*t*	*β*	*SE*	*t*
Sex	0.061	0.731	0.827	−0.038	0.067	−0.574	0.255	0.059	4.305 ***
Age	−0.435	0.373	−1.164	0.015	0.341	0.046	0.078	0.302	0.258
Grade 1	0.284	0.392	0.725	0.005	0.358	0.015	−0.015	0.317	−0.046
Grade 2	0.715	0.760	0.940	−0.099	0.695	−0.142	−0.073	0.615	−0.117
PEI	0.202	0.037	5.503 ***	0.159	0.034	4.654 ***	0.117	0.031	3.824 ***
SC				0.392	0.034	11.526 ***	0.458	0.033	13.969 ***
CFC							0.191	0.033	5.797 ***
R^2^	0.048			0.207			0.379		
F	7.252 ***			31.227 ***			62.685 ***		

Note: *** *p* < 0.001.

**Table 3:** Analysis of Mediating Effects Between Variables.

Path		Effect	Boot SE	95% CI		Relative Mediation Effect
				LLCI	ULCI	
Direct effect	PEI → ADG	0.117	0.031	0.057	0.178	45.88%
Indirect effect	PEI → SC → ADG	0.092	0.020	0.054	0.133	36.08%
	PEI → CFC → ADG	0.030	0.009	0.015	0.050	11.76%
	PEI → SC → CFC → ADG	0.015	0.004	0.008	0.024	5.88%
Total indirect effect		0.138	0.023	0.094	0.183	54.07%
Total effect		0.255	0.036	0.185	0.326	100.00%

## Data Availability

The data that support the findings of this study are available from the corresponding author upon reasonable request.
